# Pleiotropy facilitates parallel adaptation in sticklebacks

**DOI:** 10.1111/mec.16335

**Published:** 2022-01-22

**Authors:** Diana J. Rennison, Catherine L. Peichel

**Affiliations:** ^1^ Division of Evolutionary Ecology Institute of Ecology and Evolution University of Bern Bern Switzerland; ^2^ Present address: Division of Biological Sciences University of California at San Diego La Jolla California USA

**Keywords:** adaptation, parallel evolution, pleiotropy, population genomics, quantitative trait loci, weighted correlation network analysis

## Abstract

Highly pleiotropic genes are predicted to be used less often during adaptation, as mutations in these loci are more likely to have negative fitness consequences. Following this logic, we tested whether pleiotropy impacts the probability that a locus will be used repeatedly in adaptation. We used two proxies to estimate pleiotropy: number of phenotypic traits affected by a given genomic region and gene connectivity. We first surveyed 16 independent stream‐lake and three independent benthic‐limnetic ecotype pairs of threespine stickleback to estimate genome‐wide patterns in parallel genomic differentiation. Our analysis revealed parallel divergence across the genome; 30%–37% of outlier regions were shared between at least two independent pairs in either the stream‐lake or benthic‐limnetic comparisons. We then tested whether parallel genomic regions are less pleiotropic than nonparallel regions. Counter to our a priori prediction, parallel genomic regions contained genes with significantly more pleiotropy; that is, influencing a greater number of traits and more highly connected. The increased pleiotropy of parallel regions could not be explained by other genomic factors, as there was no significant difference in mean gene count, mutation or recombination rates between parallel and nonparallel regions. Interestingly, although nonparallel regions contained genes that were less connected and influenced fewer mapped traits on average than parallel regions, they also tended to contain the genes that were predicted to be the most pleiotropic. Taken together, our findings are consistent with the idea that pleiotropy only becomes constraining at high levels and that low or intermediate levels of pleiotropy may be beneficial for adaptation.

## 1 INTRODUCTION

The biological world is spectacularly diverse, and one of the most fundamental properties of organisms is their remarkable ability to evolve. Upon a cursory glance, it appears that the capacity of an organism to evolve is boundless, and the process of adaptation to new environments is relatively stochastic. However, work over the last decades has provided tantalizing clues that evolutionary trajectories may be more constrained and deterministic than previously appreciated (reviewed by Bolnick et al., 2018). Knowing whether evolution is predictable or stochastic is of fundamental importance in evolutionary biology and has critical implications for the fields of agricultural breeding, conservation, and medicine. Yet, we are far from understanding the factors that underlie the predictability of evolutionary responses.

The repeated evolution of similar phenotypes in response to similar environments (hereafter referred to as parallel evolution) provides a remarkable opportunity to identify the sources of evolutionary constraints during adaptation. In particular, if the genetic basis of adaptive phenotypic changes is the same in independently evolved populations or species, it suggests that there could be underlying constraints in the types of loci used during evolution. Recent studies have demonstrated that the same genes or genomic loci are often identified when independent populations have evolved the same phenotypes or adapted to similar habitats (Conte et al., 2012; Martin & Orgogozo, 2013; Stern, 2013). Yet, these studies have not explicitly addressed the factors responsible for these patterns in nature.

Pleiotropy, the influence of a single locus on multiple traits, is thought to be an important mechanism of evolutionary constraint and could be an important deterministic factor during adaptive evolution (Martin & Orgogozo, 2013; Stern & Orgogozo, 2008). Theoretical models (Fisher, 1930; Orr, 2000; Otto, 2004) and work in quantitative genetics (Mckay et al., 2003) suggest that pleiotropy strongly impacts the probability that a given locus will be used in adaptation. Specifically, high levels of pleiotropy are predicted to decrease the frequency with which a locus is used over the course of evolution because it is much more likely that mutations in that locus will have negative fitness consequences. Conversely, loci capable of evoking the same phenotypic change but that have low levels of pleiotropy would be predicted to be used more frequently when the same phenotype evolves in parallel. Consistent with this prediction, “hotspots” of parallel genetic evolution are often found in genes or mutations that minimize pleiotropy while maximizing phenotypic change (Martin & Orgogozo, 2013; Stern & Orgogozo, 2008). However, there is also the possibility for synergistic pleiotropy, when a mutation or gene improves two or more traits (Leiby & Marx, 2014), which may circumvent the theorized cost of pleiotropy. Synergistic pleiotropy has even been suggested to drive adaptive evolution (e.g., Frachon et al., 2017; McGee et al., 2016). Despite these theoretical predictions and insights from specific case studies, we lack comprehensive and unbiased tests of how pleiotropy contributes to evolutionary predictability.

Here, we provide such a test of the contribution of pleiotropy to parallel adaptation at the genomic level. Pleiotropy is notoriously difficult to directly estimate because it requires a thorough characterization of all of the traits affected by a given gene in a particular environment. While researchers have direct estimates of the pleiotropic effects of a handful of genes on phenotypic evolution (e.g., Mills et al., 2014; Greenwood et al., 2016; Smith, 2016; Nagy et al., 2018; Lewis et al., 2019; Archambeault et al., 2020), we lack these estimates genome‐wide except in a few model organisms where phenotypes have been measured under laboratory conditions (Wang et al., 2010). We sought to overcome these difficulties using two proxies for pleiotropy. First, we used number of phenotypic traits affected by a given genomic region, which was determined from quantitative trait locus (QTL) mapping data, because evidence of multiple traits mapping to the same locus provides evidence of pleiotropy (Wagner & Zhang, 2011). Second, we used gene connectivity, which was estimated from a gene coexpression network, as a metric because mutational screens and network analyses have previously suggested that highly connected genes exhibit greater pleiotropy (Featherstone & Broadie, 2002; He & Zhang, 2006; Wagner et al., 2007). Network connectivity can indicate gene regulatory interactions, protein‐protein interactions, or position in metabolic pathways (Proulx et al., 2005). Furthermore, gene connectivity has been found to be an important determinant of evolutionary constraint when responding to a selective challenge; genes with higher levels of connectivity tend to evolve at a slower rate (Mähler et al., 2017; Masalia et al., 2017) and are associated with signatures of local adaptation (Hämälä et al., 2020). By using two different proxies, we were able to evaluate whether the observed relationship between genomic parallelism and pleiotropy was robust to the metric used.

Genetic factors aside from pleiotropy, that is, recombination rate and mutation rate, have also been hypothesized to contribute to patterns of parallel genomic evolution, so we also considered their effects here. When populations are experiencing strong divergent selection in the presence of gene flow, genetic differentiation is strongly biased towards the genomic regions with the lowest recombination rates (Samuk et al., 2017). This reduction in the fraction of the genome that is able to maintain loci under divergent selection could lead to an increased probability of parallel evolution in genomic regions with suppressed recombination. Mutation rate might also influence the probability of parallel evolution. High mutation rates may frequently generate mutations, allowing independent populations to acquire the same or similar adaptive mutations, as seen in the parallel loss of the pelvic girdle in threespine stickleback (Xie et al., 2019).

The threespine stickleback (*Gasterosteus aculeatus*) is an ideal system to test whether pleiotropy is a source of evolutionary constraint. These small fish are a classic example of parallel evolution, as they have independently and repeatedly adapted to freshwater habitats in the northern hemisphere since the retreat of the glaciers 12,000 years ago (Bell & Foster, 1994). Stickleback living in similar habitats have evolved similar phenotypes, with phenotypic divergence between sticklebacks inhabiting divergent habitats. One widespread example is divergence between the stickleback found in streams versus lakes. These stream‐lake pairs exhibit patterns of repeatable phenotypic evolution across Canada and Europe (Berner et al., 2008; Lucek et al., 2013; Stuart et al., 2017). Repeated phenotypic evolution is also found in three lakes in British Columbia, Canada where benthic and limnetic morphs have evolved repeatedly in sympatry (Gow et al., 2008; Schluter & McPhail, 1993) and exhibit considerable genetic and phenotypic parallelism (Conte et al., 2015; Jones, Chan, et al., 2012). Here, we used these two independent sets of stickleback species pairs along with two proxies of pleiotropy to investigate the genetic factors that contribute to the repeatability of genomic differentiation. In each species‐pair comparison we first determined the genomic regions diverging in parallel across our replicate population pairs (16 independent stream‐lake pairs and 3 independent benthic‐limnetic pairs). We define parallel regions as those that are outliers in the genome‐wide *F*_ST_ distribution in at least two independent population pairs of an ecotype (stream‐lake or benthic‐limnetic). We then used these genome‐wide data to test the predictions that the parallel loci used repeatedly for adaptation have lower levels of predicted pleiotropy, are in regions of low recombination, or have higher mutation rates.

## 2 MATERIALS AND METHODS

### 2.1 Quantification of parallelism

Illumina sequence data for 16 independent pairs of stream and lake stickleback (32 populations, 24 individuals per population) from British Columbia, Canada was previously generated by (Stuart et al., 2017), using the double‐digest restriction‐site associated DNA‐sequencing (double‐digest RAD) method. Illumina sequence data for the three independent pairs of benthic and limnetic stickleback (6 populations, 20 individuals per population) from British Columbia, Canada was previously generated by (Samuk et al., 2017) using the genotyping‐by‐sequencing (GBS) method. Single nucleotide polymorphisms (SNPs) were identified using a standard, reference‐based bioinformatics pipeline (see Samuk et al., 2017 for full details). For both data sets, alignment of reads was done to the *Gasterosteus aculeatus*.BROADS1.97 genome assembly (Jones, Grabherr, et al., 2012) for consistency with prior analyses of the same data sets (Samuk et al., 2017; Stuart et al., 2017).

Weir‐Cockerham Fixation index (*F*_ST_) (Weir & Cockerham, 1984) was used to estimate genetic differentiation for each independent pair of stream‐lake or benthic‐limnetic populations, equating to 19 independent genome‐wide *F*_ST_ data sets. *F*_ST_ was chosen as the metric of differentiation rather than *D_xy_* because it is less affected by the incomplete lineage sorting, which is widespread in stickleback (Samuk et al., 2017). Window‐averaged *F*_ST_ values were calculated across 50 kilobase pair (kbp) windows by dividing the sum of the numerators of all SNP‐wise *F*_ST_ estimates within a given window by the sum of their denominators. These windows were constrained to have the same size and genomic location for each stream‐lake or benthic‐limnetic population pair. For downstream analysis we required that each window contained at least three variable sites. Genomic windows were classified as “outliers” or “nonoutliers” based on their mean *F*_ST_. We classified outlier windows as those with mean *F*_ST_ values falling within the top 5% of the genome‐wide *F*_ST_ distribution within a given stream‐lake or benthic‐limnetic comparison. Outlier classification was performed using custom R scripts (archived in Dryad doi: https://doi.org/10.5061/dryad.s4mw6m97r) (R version 3.6.0) (R Core Development Team, 2019). There were a total of 2,513 windows meeting the criteria for inclusion in the stream‐lake analysis (previously reported and analysed in Rennison et al., 2019) and 5,733 windows for the benthic‐limnetic analysis. A window was categorized as parallel if it was an outlier in two or more independent population pairs of a given ecotype contrast (i.e., stream‐lake or benthic‐limnetic). Because there are only three independent pairs of benthic‐limnetic ecotypes, we chose two as the cutoff for parallelism in both data sets; this allowed us to include windows in which data was only available for two independent benthic‐limnetic pairs. A window was categorized as nonparallel if it was not an outlier in any population pair or if it was an outlier in only a single population pair. For the results presented in the main paper, we compared our proxies of pleiotropy between parallel and nonparallel windows (see below). However, the results of similar comparisons between parallel outlier versus nonparallel outlier windows, as well as between outlier versus not outlier windows are presented in Supporting Information.

In addition to the comparisons of outlier vs. not outlier windows, we also performed analyses based on continuous values of *F*_ST_. For these analyses, it was necessary to normalize *F*_ST_ across the independent population pairs, as the replicate pairs varied in the overall magnitude of divergence. To normalize the *F*_ST_ values we determined the rank value of *F*_ST_ for each window in the *F*_ST_ distribution using the “rank” command in R, with the “ties.method” set to “average”. This method assigned larger rank values to windows with higher *F*_ST_ values and smaller rank values to windows with lower *F*_ST_ values. All windows with the same *F*_ST_ value received the same rank value. We then averaged the rank scores for each window across the comparisons for each ecotype (the 16 stream‐lake pairs or the 3 benthic‐limnetic pairs).

### 2.2 Quantification of QTL trait number and PVE

The stickleback QTL database assembled by (Peichel & Marques, 2017) was used to determine the total number of traits mapped to each 50 kbp genomic region. Only traits mapped in threespine stickleback (*G*. *aculeatus*) were used. The database was curated to ensure that if a QTL for the same trait mapped to the same window, it was only counted one time (see Supporting Information Materials and Methods for full curation details). In the final data set (Data S1) there were 858 QTL for 219 traits across eight trait categories that diverge among stream‐lake and benthic‐limnetic pairs: behaviour/sensory system (24 QTL for 16 traits, e.g., lateral line sensory system, various components of schooling behaviour), body shape (312 QTL for 78 traits, e.g., linear lengths, geometric morphometric landmarks), body size (3 QTL for 1 trait e.g., centroid size), defensive armour (135 QTL for 19 traits, e.g., anal, dorsal and pelvic spine lengths, lateral plate number and size), feeding morphology (333 QTL for 79 traits, e.g., tooth numbers, gill raker numbers and lengths, premaxilla length), pigmentation (13 QTL for 8 traits, e.g., nuptial coloration, melanophore patterning), respiration (17 QTL for 6 traits, e.g., opercle measurements), and swimming (21 QTL for 12 traits, e.g., fin ray number, vertebrae number) (Peichel & Marques, 2017). The mid‐point of the marker confidence interval was used to position each QTL into one of the predefined 50 kbp windows. If there was no information on the confidence interval, the mid‐point of the flanking markers was used. Once traits were assigned to windows, the mean percent variance explained (PVE) of the QTL for all mapped traits was estimated from the values in the database. If independent studies mapped the same trait to the same window, PVE was averaged for that trait before estimating the mean for all traits.

### 2.3 Quantification of connectivity

RNA‐seq data previously generated by Huang et al. (2016) from the spleens and head kidneys of stream and lake threespine stickleback was used to build a gene coexpression network, which allowed the estimation of total connectivity for each gene. Raw RNA‐seq reads were downloaded from the European nucleotide archive on 5 July, 2019. Reads were aligned to the *Gasterosteus aculeatus*.BROADS1.97 transcript reference (Jones, Grabherr, et al., 2012). Paired‐end read mapping was done using STAR 2.6.0c (Dobin et al., 2013). Gene expression was estimated using RSEM 1.3.0 with the default parameters (Li & Dewey, 2011). A custom PERL script (archived in Dryad doi: https://doi.org/10.5061/dryad.s4mw6m97r) was used to output FPKM and create the final expression table. FPKM values were log transformed (log2(x+1)) according to (Langfelder & Horvath, 2008). Loci whose variance fell below the first quantile of genome‐wide variance were dropped for the data set as these lowly expressed or nonvarying genes usually represent noise (Langfelder & Horvath, 2008).

The WGCNA R package for weighted correlation network analysis (Langfelder & Horvath, 2008) was used to build a gene coexpression network from the final gene expression data. A soft‐thresholding power was selected after visual inspection of plots of the network topology analysis. At a power of 16 we saw saturation of the scale free topology model fit and mean connectivity and thus selected this value for our network construction. We used the “blockwiseModules” function to build our network, using an unsigned network and maximum block size of 20,000 (which allowed all loci to be estimated in a single block). The minimum module size used in the final network was 30, merged cut height was 0.25 and reassignment threshold was 0. However, increasing or decreasing each parameter by 50% did not affect any of the connectivity results reported in the paper. Intramodular connectivity was estimated using the “intramodularConnectivity.fromExpr” function. We used the “cor” correlation function and the Euclidean distance option. We then extracted the total connectivity values (kTotal), which is the connectivity of a gene within and between modules for each gene. The mid‐point of the coding region of a gene was used to match the estimates of total connectivity to the predefined 50 kbp windows. Connectivity values were averaged across all the genes that mapped to a given window, and these mean connectivity values were used in the downstream analysis.

### 2.4 Caveats of pleiotropy metrics

We chose to estimate pleiotropy using two independent metrics because we could not estimate pleiotropy directly. One limitation to the use of QTL mapping data for estimating pleiotropy is that we cannot tell whether observed comapping of independent traits is due to pleiotropy or linkage. A second limitation of the QTL data is that we do not know whether the direction of effects of the QTL on phenotypes lead to synergistic or antagonistic effects on fitness. A third limitation is that we rely on QTL data collected from a variety of different stickleback populations. Although more than half of the QTL are from crosses involving two of the benthic populations studied here and involve many traits known to evolve in parallel in benthic‐limnetic pairs (Conte et al., 2015), only 16% of the QTL (*n* = 139) are from benthic‐limnetic crosses and less than 2% of the QTL (*n* = 17) are from a single stream‐lake cross from Europe. Thus, we do not always know whether the QTL contained within the parallel windows are divergent in our benthic‐limnetic and/or stream‐lake populations.

The connectivity data also has some key limitations. First, the connectivity data is measured at the gene level and yields higher resolution data than the QTL data, but it does not have a direct link to phenotypes or to fitness. Second, the gene coexpression network was only built from RNA‐seq data derived from stream‐lake stickleback ecotypes. Thus, it is unclear whether the observed significant positive relationship between gene connectivity and parallelism for stream‐lake pairs (and/or lack of significance for benthic‐limnetic pairs) was due to a bias created by building the gene network from only one ecotype pair. However, it is important to note that the data was from European stream‐lake populations, which are very distantly related to the Canadian stream‐lake populations used here and exhibit very little genetic parallelism (Rennison et al., 2020). Despite the different biases and limitations of these two metrics, when taken together they provide complementary (i.e., phenotype‐dependent and phenotype‐independent) estimates of pleiotropy (Hämälä et al., 2020).

### 2.5 Estimation of mutation rate, gene density and recombination

We utilized previous estimates of mutation rate and recombination rate (Samuk et al., 2017). The recombination rates (cM/Mbp) were estimated from a high‐density genetic map (Roesti et al., 2013). Mutation rates were estimated using PAML (Yang, 2007) as the synonymous substitution rate (dS) across a four species phylogeny (see Samuk et al., 2017 for a full description of the methods used to estimate these two variables). Gene density was estimated using the package BioMart to obtain a list of all annotated *G*. *aculeatus* coding DNA sequences from ENSEMBL (with start and end positions). We then used a custom R script (archived in Dryad doi: https://doi.org/10.5061/dryad.s4mw6m97r) to estimate mean recombination rate, mean mutation rate, and total gene number for each of the pre‐defined 50 kbp windows.

### 2.6 Comparison of pleiotropy, mutation rate, recombination rate and gene density between parallel and nonparallel windows

Using custom R scripts (archived in dryad doi: https://doi.org/10.5061/dryad.s4mw6m97r), we estimated the empirical difference in proxies of pleiotropy between parallel and nonparallel windows using a linear model. Since pleiotropy estimates were non‐normally distributed, we used permutations with 10,000 iterations to build a null distribution to test for significance. For each iteration, the status of a given window as parallel or nonparallel was randomly shuffled across the genome, with missing data held in place. Then with the parallelism status of all windows randomized, we re‐estimated the magnitude of difference in the level of pleiotropy between parallel and nonparallel windows. We repeated the process 10,000 times, which yielded a null distribution of the difference in pleiotropy. We then compared our empirical estimate against this null to determine statistical significance; these are the *p*‐values reported in the manuscript. The same process was repeated to estimate the significance of the differences in mutation rate, recombination rate, and gene density between parallel and nonparallel windows. Linear models were used to determine the strength of the relationship between our continuous metric of differentiation (mean *F*_ST_ rank) and our parallelism metrics (number of mapped traits or connectivity), as well as recombination rate, mutation rate, and gene density.

## 3 RESULTS

### 3.1 Levels of parallelism across the genome

Mean genome wide *F*_ST_ values ranged from 0.03 to 0.18 for stream‐lake pairs and 0.19 to 0.21 for benthic‐limnetic pairs (Table S1). In a previous analysis of genomic data from 16 independent stream‐lake population pairs (Rennison et al., 2019), we found 15% of all 50 kbp windows (37% of outlier windows) were *F*_ST_ outliers shared by at least two independent stream‐lake population pairs, with up to ten pairs sharing an outlier window (although most are shared by two to four pairs) (Figure S1). For the benthic‐limnetic population pairs, 3% of all genomic windows (30% of outlier windows) were *F*_ST_ outliers shared by at least two independent pairs, with 7% of outlier windows shared among all three of the pairs (Figure S1).

### 3.2 Comparison of pleiotropy between parallel and nonparallel windows

**FIGURE 1:**
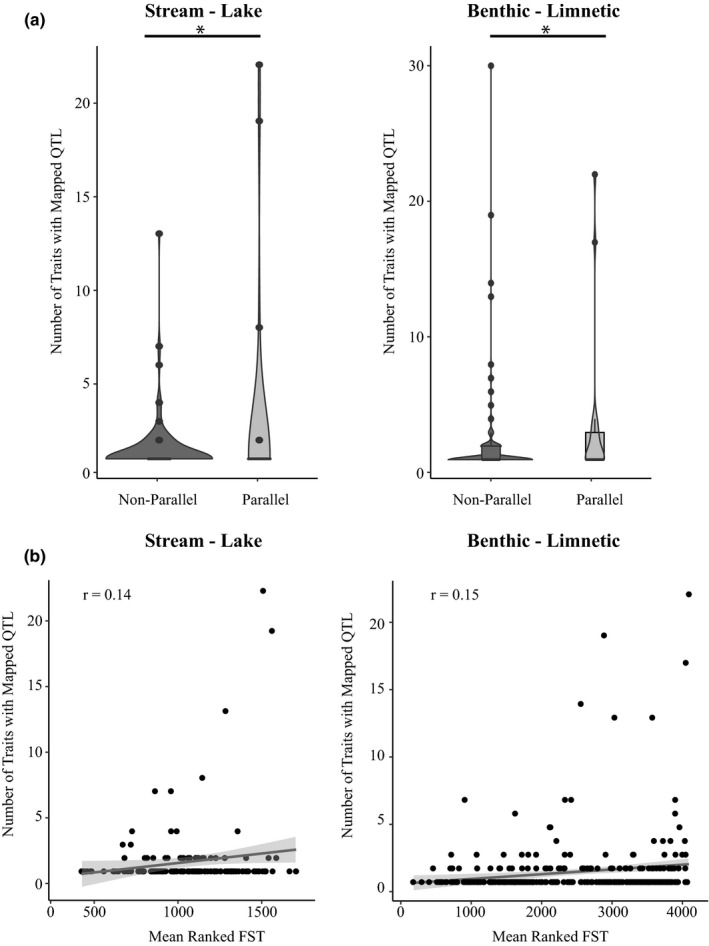
(a) Comparisons of pleiotropy (number of traits with mapped QTL) between parallel and nonparallel windows. An asterisk indicates permutation test *p* < .05. (b) Relationship between pleiotropy (number of traits with mapped QTL) and mean *F*_ST_ rank. Note that the total number of windows with data or having unique *F*_ST_ values differed between the stream‐lake and benthic‐limnetic ecotype pairs so that the mean *F*_ST_ rank values differ among these comparisons

Pleiotropy in each genomic window was estimated using two independent proxies, number of mapped QTL (measured as either total number of traits affected by the QTL or mean PVE of all those QTL) and mean gene connectivity. All pleiotropy proxies yielded estimates with exponential frequency distributions across the windows (Figure S2). Using the QTL estimates of pleiotropy, we found that parallel windows contained twice as many mapped traits as nonparallel windows (stream‐lake: parallel windows = 2.96 ± 1.10 traits, nonparallel windows = 1.49 ± 0.11 traits, permutation test *p* = .004; benthic‐limnetic: parallel windows = 3.48 ± 1.20 traits, nonparallel windows = 1.68 ± 0.11, permutation test *p* = .002) (Figure 1a). QTL in parallel windows also tended to explain more variance than those in nonparallel windows, although this was not quite significant in the stream‐lake comparison (stream‐lake: parallel windows = 13.68 ± 4.14%, nonparallel windows = 9.48 ± 0.95%, permutation test *p* = .074; benthic‐limnetic: parallel windows = 17.36 ± 4.7%, nonparallel windows = 9.02 ± 0.48%, permutation test *p* = .0002) (Figure S3). The pattern of significantly increased pleiotropy in parallel windows relative to nonparallel windows remains true if only windows that were an outlier in at least one population are considered; we also find no difference in the number of QTL mapping to outlier windows compared to nonoutlier windows (see Supporting Information text for full statistical results).

To avoid any possible biases imposed by categorizing windows into the binary classes of parallel or nonparallel, we also used a continuous metric of repeatability: mean *F*_ST_ rank across replicate ecotype pairs (a larger rank indicates that a window had a higher *F*_ST_ value among the distribution of values in a given population pair; thus a larger mean rank indicates a parallel window that had a higher *F*_ST_ value across multiple pairs). Using mean *F*_ST_ rank, we find that for both the benthic‐limnetic and stream‐lake comparisons, genomic windows with higher mean *F*_ST_ rank contain significantly more mapped QTL (stream‐lake correlation coefficient (*r*) = 0.14, *p* = .032; benthic‐limnetic *r* = 0.15, *p* = .0022) (Figure 1b).

**FIGURE 2:**
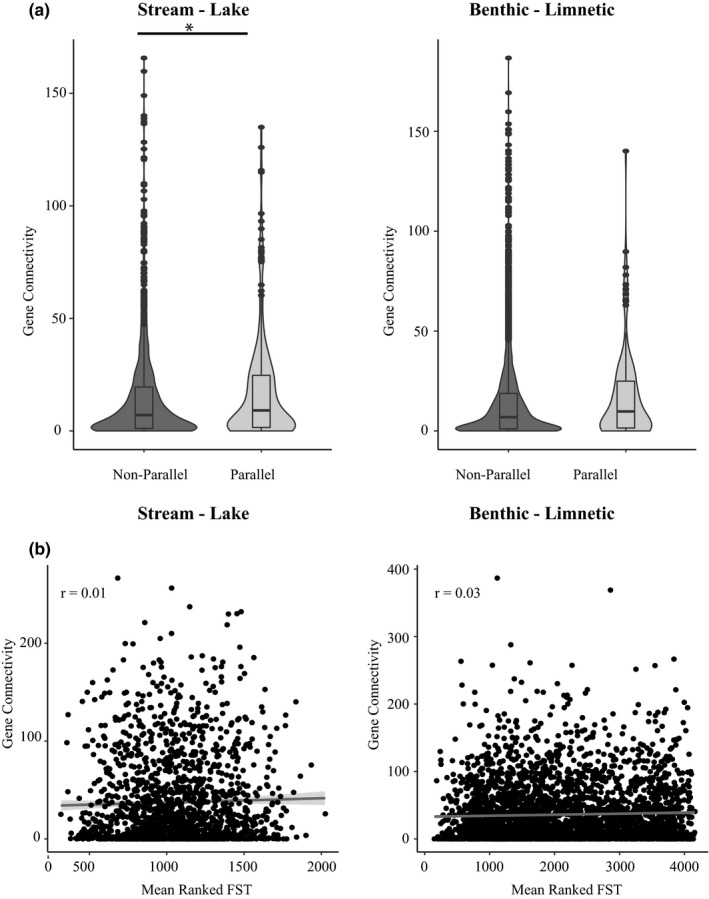
(a) Comparisons of pleiotropy between parallel and nonparallel windows as measured by mean connectivity. An asterisk indicates permutation test *p* < .05. (b) Relationship between mean connectivity and mean *F*_ST_ rank. Note that the total number of windows with data or having unique *F*_ST_ values differed between the stream‐lake and benthic‐limnetic ecotype pairs so that the mean *F*_ST_ rank values differ among these comparisons

Using gene connectivity as the metric of pleiotropy we find the same pattern of increased pleiotropy in parallel windows and in windows with higher mean *F*_ST_ rank. Genes in parallel windows were 1.2‐fold more connected than those in nonparallel windows (stream‐lake: parallel windows = 18.1 ± 1.5, nonparallel windows = 14.9 ± 0.6 mean connectivity, permutation test *p* = 0.014; benthic‐limnetic: parallel windows = 18.1 ± 1.7, nonparallel windows = 14.2 ± 1.0 mean connectivity, permutation test *p* = .063) (Figure 2a). There is also a positive relationship between gene connectivity in a window and the mean *F*_ST_ rank across replicate stream‐lake (*r* = 0.01, *p* = .13) and benthic‐limnetic ecotype pairs (*r* = 0.03, *p* = .03) (Figure 2b). The relationship between gene connectivity and parallelism did not change if only outlier windows were considered, and connectivity levels did not differ between outlier and nonoutlier windows (see Supporting Information text for full statistical results).

### 3.3 Comparison of gene density, mutation rate, and recombination rate between parallel and nonparallel windows

**FIGURE 3:**
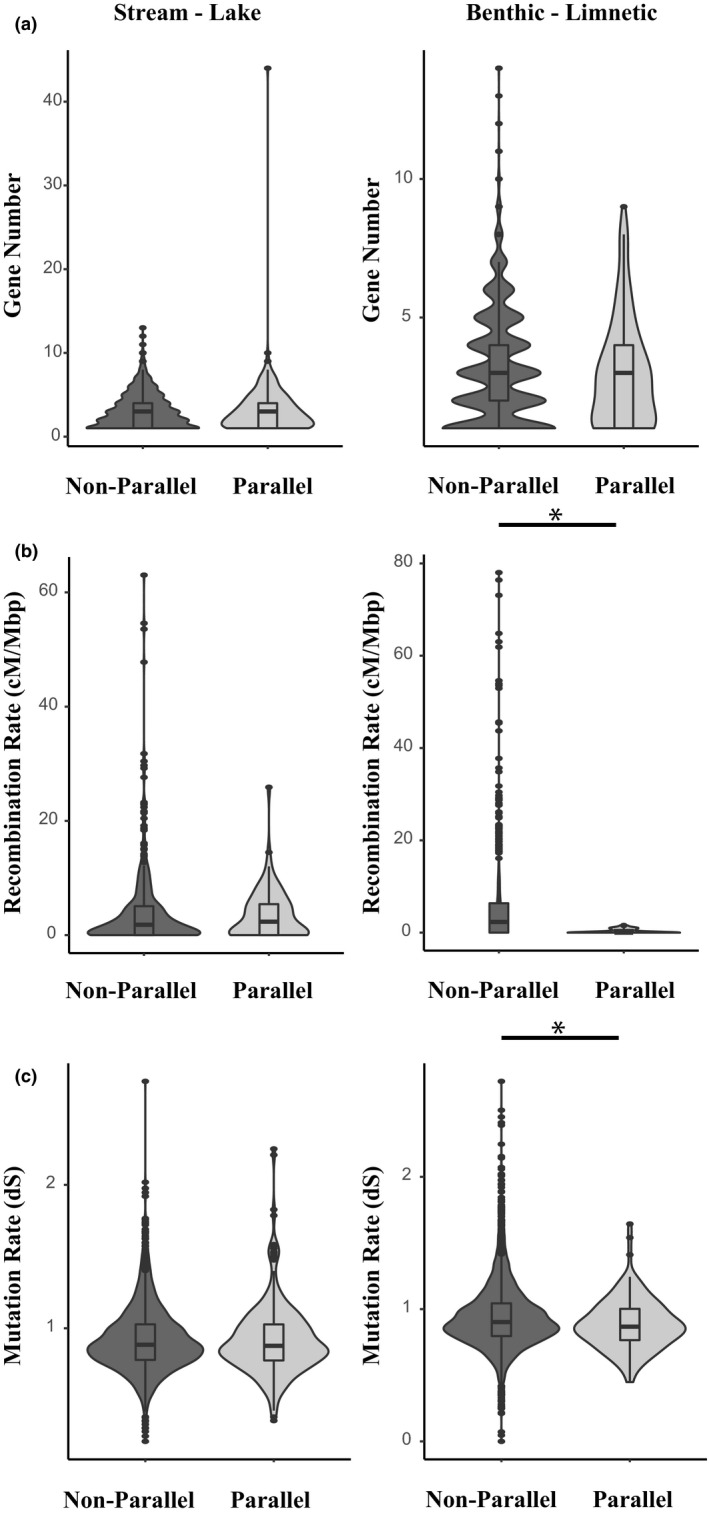
Comparison of (a) gene number, (b) recombination rate (cM/Mbp), and (c) mutation rate (dS) between parallel and nonparallel windows. An asterisk indicates permutation test *p* < .05

There was no significant difference in the mean gene count between parallel and nonparallel windows (stream‐lake: parallel windows = 3.23 ± 0.2 genes, nonparallel windows = 3.1 ± 0.1 genes, permutation test *p* = .18; benthic‐limnetic: parallel windows = 3.0 ± 0.2 genes, nonparallel windows = 3.2 ± 0.03 genes, permutation test *p* = .85) (Figure 3a). In the stream‐lake comparison, there was no difference in mean recombination rate (parallel windows = 3.6 ± 0.51 cM/Mbp, nonparallel = 4.24 ± 0.34 cM/Mbp, permutation test *p* = .76) or mean mutation rates (parallel windows dS = 0.92 ± 0.02, nonparallel windows dS = 0.92 ± 0.01, permutation test *p* = .45) between parallel and nonparallel windows (Figure 3b,c). In the benthic‐limnetic comparison, mean recombination and mutation rates were significantly lower for parallel windows (recombination: parallel windows = 0.30 ± 0.1 cM/Mbp, nonparallel windows = 4.86 ± 0.2 cM/Mbp, permutation test *p* = .002; mutation rate (dS): parallel windows = 0.89 ± 0.017, nonparallel windows = 0.94 ± 0.003, permutation test *p* = .004) (Figure 3b,c). Recombination rates and mutation rates were positively correlated (*r* = 0.12, *p* = .0001) in the benthic‐limnetic comparison. However, when only outlier windows were considered in the benthic‐limnetic comparison, there was no significant difference in the recombination or mutation rate of parallel versus nonparallel windows (recombination rate: nonparallel outlier windows = 2.45 ± 0.8 cM/Mbp, parallel outlier windows = 0.30 ± 1.6 cM/Mbp, permutation test *p* = .09; Mutation rate (dS): nonparallel outlier windows = 0.90 ± 0.01, parallel outlier windows = 0.89 ± 0.02, permutation test *p* = .32). The analyses using our continuous estimate of parallelism, rank *F*_ST_ showed the same patterns (see Figure S4).

## 4 DISCUSSION

### 4.1 Magnitude of parallelism

Benthic‐limnetic pairs exhibit a relatively high magnitude of parallelism (33%) among putatively adaptive loci; this matches the findings of a previous study where ~33% of benthic‐limnetic outlier SNPs were shared among at least two pairs (Jones, Chan, et al., 2012). A similar level of parallelism was observed for stream‐lake pairs (37% of outliers), with two to 10 population pairs sharing a given outlier. The variable magnitude of parallelism observed for stream‐lake pairs mirrors phenotypic parallelism, which has also been shown to be highly variable in these populations (Stuart et al., 2017).

### 4.2 Association between parallelism and pleiotropy

Parallel windows contain loci that are more pleiotropic on average than loci in windows that are adaptive in only a single population pair or evolving neutrally. This pattern was consistent across the two ecotype pairs (benthic‐limnetic and stream‐lake) and across the two measures of pleiotropy (number of mapped QTL and gene connectivity). Both pleiotropy proxies were found to have exponential distributions, suggesting that most genes have low levels of predicted pleiotropy, while some genes are highly pleiotropic. Our distributions of pleiotropy were similar to those found previously for yeast, nematodes and mice (Wang et al., 2010).

On average, nonparallel windows contained genes that were less connected and influenced fewer mapped traits than parallel windows. However, we observed that nonparallel windows also tended to contain the genes that were the most pleiotropic (i.e., most connected and influencing the greatest number of traits) (i.e., Figure 1, Figure 2, and Figure S3). To examine the possibility that intermediate levels of pleiotropy are favoured in parallel windows, we examined the relationship between the magnitude of pleiotropy and the magnitude of parallelism. Unfortunately, there was not enough variation in magnitude of parallelism to conduct these analyses using the three available benthic‐limnetic pairs. However, in the stream‐lake pairs, the greatest levels of pleiotropy are seen at intermediate levels of parallelism, when two or three populations share an outlier region (Figure S5). These data suggest that higher levels of pleiotropy might become constraining.

Overall, a pattern of increased pleiotropy in parallel outlier windows is consistent with the idea that low or intermediate levels of pleiotropy may be beneficial for adaptation (Frachon et al., 2017; Wagner & Zhang, 2011; Wang et al., 2010), and that it is only at high levels that pleiotropy becomes constraining (Hansen, 2003). These recent findings conflict with earlier theoretical work suggesting that pleiotropy is constraining and should always be disfavoured (Fisher, 1930; Orr, 2000; Otto, 2004). Our results are also contrary to the suggestion that genes and mutations that minimize pleiotropy will contribute more often to repeated phenotypic evolution (Martin & Orgogozo, 2013; Stern & Orgogozo, 2008), but consistent with more recent work demonstrating that pleiotropic loci can contribute to adaptive phenotypic evolution (Archambeault et al., 2020; Greenwood et al., 2016; Hämälä et al., 2020; Lewis et al., 2019; Mills et al., 2014; Nagy et al., 2018; Smith, 2016).

Why might pleiotropic loci be reused over the course of adaptive evolution? First, low levels of pleiotropy can increase the genetic variation available for selection to work upon (Hansen, 2003). Second, synergistic pleiotropy can facilitate a rapid change of multiple characters simultaneously, allowing coadaptation of a suite of traits (Frachon et al., 2017; Wagner & Zhang, 2011; Wang et al., 2010). Loci with such synergistic pleiotropic effects might be particularly maintained as standing genetic variation in systems such as sticklebacks, in which repeated adaptation to similar environments has occurred many times (Jones, Grabherr, et al., 2012; Nelson & Cresko, 2018). These bouts of recurrent selection might lead to the preservation of genetic variants with optimal levels of pleiotropy. Future work should determine whether adaptive loci arising due to standing genetic variation have higher levels of pleiotropy than those due to novel mutation. Most crucially, future work should also determine whether traits with a pleiotropic genetic basis have synergistic or antagonistic fitness effects. Such studies will provide insight into the mechanisms by which pleiotropy is not a constraint and perhaps even beneficial.

### 4.3 Association between parallelism and other genomic factors

It is important to note that the observed increase in mean pleiotropy of parallel windows could not be explained by other genomic factors. We did find that in the benthic‐limnetic comparison, mean recombination and mutation rates were significantly lower for parallel windows. However, in the benthic‐limnetic pairs recombination rates and mutation rates were positively correlated, which probably accounts for the observed reduced mutation rates in parallel windows. The finding of low recombination rates in parallel windows for benthic‐limnetic stickleback is not completely surprising. It has previously been shown that there is a strong relationship between low recombination rate and probability of a variant being classified as an outlier in stickleback populations that are experiencing ongoing gene flow paired with strong divergent selection (Samuk et al., 2017). It is known that there is ongoing gene flow between benthic and limnetic stickleback in all three pairs (D. Rennison, unpublished data). The absence of a relationship between recombination rate and outlier status (or parallelism) for stream‐lake stickleback pairs may be explained by lower levels of gene flow between these ecotypes (Samuk et al., 2017; Stuart et al., 2017) or perhaps by weaker divergent selection. The effect of recombination in benthic‐limnetic pairs could also be explained by some other shared feature of the genome that is correlated with recombination rate. Nonetheless, the effect of recombination in these pairs cannot fully explain the association between parallelism and pleiotropy because it is still found when only outlier windows are considered. Thus, pleiotropy appears to be a key predictor of patterns of repeatable divergence in threespine stickleback.

### 4.4 Concluding remarks

In two independent systems of wild stickleback and using two independent proxies for pleiotropy, we consistently see that genomic regions diverging in parallel have higher levels of pleiotropy than nonparallel regions. We find evidence that intermediate levels of pleiotropy are favoured, suggesting that high levels of pleiotropy may become constraining. Thus, levels of pleiotropy appear to affect genome‐wide patterns of repeated divergence in nature. In contrast, parallel divergence is not readily explained by differences in mutation rate, gene density or recombination rate. This study provides further empirical evidence supporting the idea that low or intermediate levels of pleiotropy are not constraining, and that some degree of pleiotropy may actually be advantageous for rapid adaptation.

## CONFLICT OF INTEREST

The authors declare they have no conflicting interests regarding this study.

## AUTHOR CONTRIBUTIONS

Diana J. Rennison and Catherine L. Peichel conceived of the idea behind the study; Diana J. Rennison analysed the data; Diana J. Rennison wrote the manuscript with input from Catherine L. Peichel.

## Supporting information

Supplementary Material

Supplementary Material

## Data Availability

All of the input files and corresponding scripts have been archived in the Dryad repository doi: https://doi.org/10.5061/dryad.s4mw6m97r). Accession numbers for raw sequence data used in the study can be found in Table S2.
