# Supplementary material for: Pleiotropy facilitates parallel adaptation in sticklebacks
Source: Mol Ecol. 2022 Jan 22;31(5):1476–86. doi: 10.1111/mec.16335 (PMC9306781; doi:10.1111/mec.16335)
Supplement: Supplementary file 1 — Supplementary Material [file MEC-31-1476-s002.docx]

**Supplemental Information for:**

**Pleiotropy facilitates parallel adaptation in sticklebacks**

**Diana J. Rennison, Catherine, L. Peichel**

**Table of Contents:**

| **Supplementary Information Text** | Pages 1 -2 |
| --- | --- |
| **Table S1** | Page 3 |
| **Table S2** | Page 4 |
| **Figure S1** | Page 5 |
| **Figure S2** | Page 6 |
| **Figure S3** | Page 7 |
| **Figure S4** | Page 8 |
| **Figure S5** | Page 9 |

**Supplementary Information Text**

**Patterns of pleiotropy between parallel outlier vs. non-parallel outlier windows.** Using the QTL estimates of pleiotropy, we found that parallel outlier windows contained twice as many mapped traits as non-parallel outlier windows (stream-lake: parallel outlier windows = 2.96 ± 1.10 traits, non-parallel outlier windows = 1.35 ± 0.45 traits, permutation test *P* = 0.026; benthic- limnetic: parallel windows = 3.48 ± 1.20 traits, non-parallel windows = 1.75 ± 0.42, permutation test *P* = 0.022). QTL in parallel outlier windows also tended to explain more variance than those in non-parallel outlier windows in the benthic-limnetic comparison (parallel outlier windows = 17.36 ± 4.7%, non-parallel outlier windows = 9.72 ± 2.1%, permutation test *P* = 0.03) but not in the stream lake comparison (parallel outlier windows = 13.68 ± 4.14%, non-parallel outlier windows = 10.98 ± 2.28%, permutation test *P* = 0.25). Using the connectivity estimates of pleiotropy, we found that parallel outlier windows were more highly connected than non-parallel outlier windows, although the pattern was not significant in the benthic-limnetic comparison, which was also true for the analysis in the main text (stream-lake: parallel outlier windows = 18.1 ± 1.5, non-parallel outlier windows = 14.21 ± 1.02, permutation test *P* = 0.010; benthic-limnetic: parallel outlier windows = 18.1 ± 1.7, non- parallel outlier windows = 15.03 ± 1.20, permutation test *P* = 0.16)

**Patterns of pleiotropy between outlier vs. non-outlier windows**. QTL estimates of pleiotropy did not differ between outlier and non-outlier windows (stream-lake: outlier windows = 2.96 ± 1.10 traits, non-outlier windows = 1.35 ± 0.45 traits, permutation test *P* = 0.42; benthic-limnetic: outlier windows = 2.2 ± 0.31 traits, non-outlier windows = 1.67 ± 0.13, permutation test *P* = 0.08). Connectivity estimates of pleiotropy did not differ between outlier and non-outlier windows (stream-lake: outlier windows = 15.66 ± 0.73, non-outlier windows = 15.21 ± 0.66 traits, permutation test *P* = 0.67; benthic-limnetic: outlier windows = 15.67 ± 0.77 traits, non-outlier windows = 14.35 ± 0.34, permutation test *P* = 0.21).

**Quantification of QTL trait number and PVE.** The Peichel and Marques 2017 dataset was curated to ensure that if a QTL for the same trait mapped to the same window, it was only counted one time. We only used the QTL identified for traits that had been mapped in crosses between threespine stickleback populations. We specified whether these crosses were between marine and freshwater ecotypes (n=138 QTL), marine and benthic ecotypes (n=561 QTL), benthic and limnetic ecotypes (n=139 QTL), lake and stream ecotypes (n=17 QTL), or stream and limnetic ecotypes (n=3 QTL). Traits were classified into eight different categories, based on their putative function. Trait names were not standardized across all of the 26 QTL mapping studies included in the original dataset. Thus, we compared the trait measurements used in different studies and changed the trait names in the dataset to be consistent across studies. For the category of “body shape”, several studies mapped QTL associated with x- and y-coordinates of morphological landmarks and reported QTL for these traits by the x- and y-coordinate number. Although many of the morphological landmarks were consistent across studies, the x- and y- coordinate numbers were not. For consistency, the names of the morphological landmarks were used, following the nomenclature in Albert et al. 2008. If a landmark was not present in this dataset, it was named following the morphological landmark used in the original study. Similarly, for the category of “feeding”, trait names were standardized when possible, following Miller et al. 2014. Finally, in the Conte et al. 2015 study, the same trait was mapped using different approaches. Thus, we ensured that only one of the redundantly mapped QTL remained in the dataset. After curation, there were 219 unique traits and 858 QTL remaining in the dataset that could be assigned to 50 kbp windows. The curated dataset is provided in Dataset S1.

**SI References**

Albert, A. Y. K., Sawaya, S., Vines, T. H., Knecht, A. K., Miller, C. T., Summers, B. R., Balabhadra, S., Kingsley, D. M., and & Schluter, D. (2008). The genetics of adaptive shape shift in stickleback: pleiotropy and effect size. *Evolution*, 62, 76-85.

Conte, G. L., Arnegard, M. E., Best, J., Chan, Y. F., Jones, F. C., Kingsley, D. M., Schluter, D., & Peichel, C. L. (2015). Extent of QTL reuse during repeated phenotypic divergence of sympatric threespine stickleback. *Genetics*, 201, 1189-1200.

Miller, C. T. *et al.* (2014). Modular skeletal evolution in sticklebacks is controlled by additive and clustered quantitative trait loci. *Genetics*, 197, 405-420.

Peichel, C. L., & Marques, D. A. (2017). The genetic and molecular architecture of phenotypic diversity in sticklebacks. *Phil. Trans R. Soc. Lond. B*, 372, 20150486.

Table S1. Mean genome-wide, minimum and maximum F_ST_ values for each replicate stickleback pair.

| Dataset | Mean Genome-wide F_ST_ | Range of F_ST_ |
| --- | --- | --- |
| Paxton Lake benthic and limnetic pair | 0.206 | 0 – 1 |
| Priest Lake benthic and limnetic pair | 0.197 | 0 – 1 |
| Little Quarry Lake benthic and limnetic pair | 0.190 | 0 – 1 |
| Beaver stream and lake pair | 0.180 | 0 - 1 |
| Boot stream and lake pair | 0.065 | 0 – 1 |
| Comida stream and lake pair | 0.066 | 0 – 1 |
| Frederick stream and lake pair | 0.058 | 0 – 1 |
| Joe stream and lake pair | 0.050 | 0 – 1 |
| Kennedy stream and lake pair | 0.028 | 0 – 0.6 |
| Misty stream and lake pair | 0.076 | 0 – 1 |
| Moore stream and lake pair | 0.036 | 0 – 1 |
| Muchalat stream and lake pair | 0.033 | 0 – 0.4 |
| Northy stream and lake pair | 0.025 | 0 – 0.6 |
| Pachena stream and lake pair | 0.068 | 0 – 1 |
| Pye stream and lake pair | 0.089 | 0 – 1 |
| Roberts stream and lake pair | 0.072 | 0 – 1 |
| Swan stream and lake pair | 0.102 | 0 – 1 |
| Thiemer stream and lake pair | 0.047 | 0 – 1 |
| Village Bay stream and lake pair | 0.047 | 0 – 1 |

Table S2. Accession numbers or link to sequence data underlying the parallelism and gene connectivity analyses.

| Dataset | Archive Location | Access Number or Link |
| --- | --- | --- |
| Benthic and limnetic GBS sequences. | NCBI Short Read Archive | SRP107890 |
| Stream and lake ddRAD sequences. | UT Austin Corral Server | http://web.corral.tacc.utexas.edu/Stuart_2017_NatureEE_Data_Code/ |
| Stream and lake RNA sequences. | European Nucleotide Archive | PRJEB8677 |

Dataset S1 (separate file). The curated QTL dataset from Peichel and Marques (2017) is provided as a .csv file. The file contains information about the ecotype comparison used to map the QTL, the study reference, the trait name, the trait category, percent variance explained (PVE), QTL position including the 50 kbp window to which it mapped (lg_bp_group), whether the window is non-parallel (value of 0) or parallel (non-zero value of the continuous parallelism estimate), and whether the window is a non-outlier, outlier in a single population, or a parallel outlier for both the stream-lake (SL) and the benthic-limnetic (BL) comparisons. NA indicates that a window was not informative in a particular ecotype comparison.

**Fig. S1.** Number of outlier populations for each 50 kbp window in the genome.

**Fig. S2.** Frequency distributions of the (A) number of traits with mapped QTL, (B) mean percent variance explained of QTL, and (C) gene connectivity in 50 kbp windows.

**Fig. S3.** Comparisons of pleiotropy between parallel and non-parallel windows as measured by mean percent variance explained of QTL. An asterisk indicates permutation test *P* < 0.05.

**
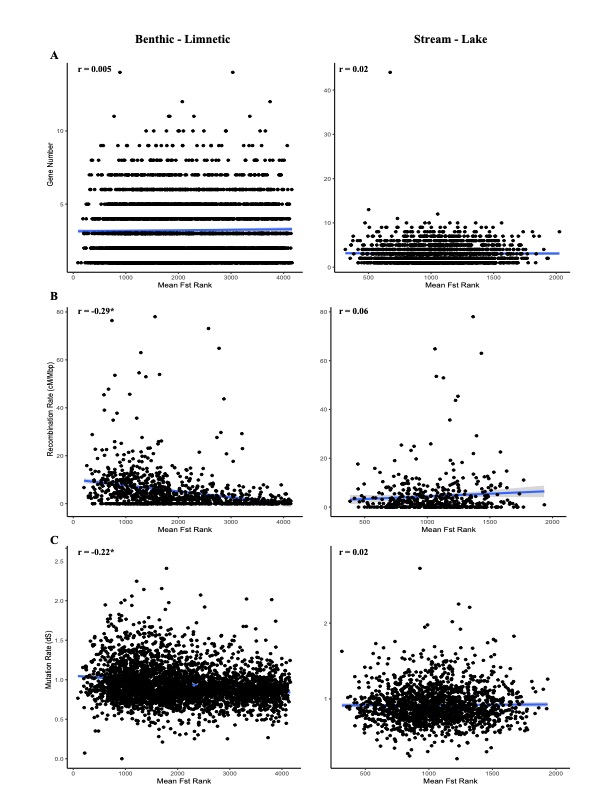
**

**Fig. S4.** Relationship between (A) gene density, (B) recombination rate, (C) mutation rate and mean *F_ST_* rank. An asterisk beside the correlation coefficient (r) indicates significance (*P* < 0.05). Note that the total number of windows with data or having unique *F_ST_* values differed between the stream-lake and benthic-limnetic ecotype pairs so that the mean *F_ST_* rank values differ among these comparisons.

**
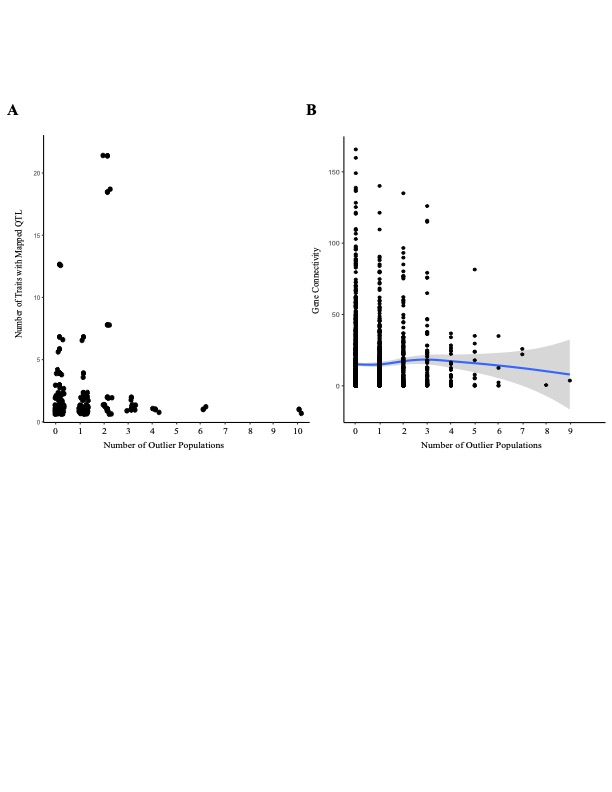
**

**Fig. S5.** Relationship between number of outlier populations and the amount of pleiotropy estimated as (A) number of traits with mapped QTL and (B) gene connectivity.
